# Mechanistic Insight into Salt Tolerance of *Acacia auriculiformis*: The Importance of Ion Selectivity, Osmoprotection, Tissue Tolerance, and Na^+^ Exclusion

**DOI:** 10.3389/fpls.2017.00155

**Published:** 2017-04-04

**Authors:** Md. M. Rahman, Md. A. Rahman, Md. G. Miah, Satya R. Saha, M. A. Karim, Mohammad G. Mostofa

**Affiliations:** ^1^Department of Agroforestry and Environment, Bangabandhu Sheikh Mujibur Rahman Agricultural UniversityGazipur, Bangladesh; ^2^Department of Agronomy, Bangabandhu Sheikh Mujibur Rahman Agricultural UniversityGazipur, Bangladesh; ^3^Department of Biochemistry and Molecular Biology, Bangabandhu Sheikh Mujibur Rahman Agricultural UniversityGazipur, Bangladesh

**Keywords:** acacia, anatomical features, halophytes, ion homeostasis, perennial species, salinity, tissue tolerance

## Abstract

Salinity, one of the major environmental constraints, threatens soil health and consequently agricultural productivity worldwide. *Acacia auriculiformis*, being a halophyte, offers diverse benefits against soil salinity; however, the defense mechanisms underlying salt-tolerant capacity in *A. auriculiformis* are still elusive. In this study, we aimed to elucidate mechanisms regulating the adaptability of the multi-purpose perennial species *A. auriculiformis* to salt stress. The growth, ion homeostasis, osmoprotection, tissue tolerance and Na^+^ exclusion, and anatomical adjustments of *A. auriculiformis* grown in varied doses of seawater for 90 and 150 days were assessed. Results showed that diluted seawater caused notable reductions in the level of growth-related parameters, relative water content, stomatal conductance, photosynthetic pigments, proteins, and carbohydrates in dose- and time-dependent manners. However, the percent reduction of these parameters did not exceed 50% of those of control plants. Na^+^ contents in phyllodes and roots increased with increasing levels of salinity, whereas K^+^ contents and K^+^/Na^+^ ratio decreased significantly in comparison with control plants. *A. auriculiformis* retained more Na^+^ in the roots and maintained higher levels of K^+^, Ca^2+^ and Mg^2+^, and K^+^/Na^+^ ratio in phyllodes than roots through ion selective capacity. The contents of proline, total free amino acids, total sugars and reducing sugars significantly accumulated together with the levels of malondialdehyde and electrolyte leakage in the phyllodes, particularly at day 150^th^ of salt treatment. Anatomical investigations revealed various anatomical changes in the tissues of phyllodes, stems and roots by salt stress, such as increase in the size of spongy parenchyma of phyllodes, endodermal thickness of stems and roots, and the diameter of root vascular bundle, relative to control counterparts. Furthermore, the estimated values for Na^+^ exclusion and tissue tolerance index suggested that *A. auriculiformis* efficiently adopted these two mechanisms to address higher salinity levels. Our results conclude that the adaptability of *A. auriculiformis* to salinity is closely associated with ion selectivity, increased accumulation of osmoprotectants, efficient Na^+^ retention in roots, anatomical adjustments, Na^+^ exclusion and tissue tolerance mechanisms.

## Introduction

Salinization of soil and groundwater has been considered as the most critical environmental issue, hindering sustainable agricultural productivity and presenting a challenging task for the environmentalists, especially in the era of global climate change. Salinity is a widespread problem, affecting around 831 million hectares of lands that include 397 and 434 million hectares of saline and sodic soils, respectively (Teakle and Tyerman, 2010). It is undesirable that every year around 1.5 million hectares of lands are being taken out of production by excessive salinity, and it has been predicted that half of the cultivable terrains will be lost due to salinity by the middle of the 21st century (Munns and Tester, 2008). High salinity causes multifarious co-operative events that adversely affect plant growth and development, and consequently yields, resulting in reduction of agricultural outputs by billions of dollars per annum (Lee et al., 2013; Song and Wang, 2015).

The rate of plant growth relies on several important events, including cell division, cell enlargement and cell differentiation, along with genetic, biochemical, physiological, morphological, and ecological processes, as well as their complex interactions, all of which can be severely disturbed by high salinity (Rahman et al., 2016). For instance, salt stress exerts osmotic stress that causes water deficit and oxidative stress. Salinity also adversely affects plant growth performance by disturbing physiological and biochemical functions involved in photosynthesis, nitrogen metabolism, ion homeostasis, antioxidant phenomena and osmolyte accumulation, leading to cell decease (Agarwal et al., 2013; Pompeiano et al., 2016). To cope with the detrimental effects of salt stress, plants have evolved intrinsic adaptation strategies that include morphological, anatomical, biochemical, and physiological changes to assist their survival (Dawood et al., 2014). These changes can be achieved through adoption of mechanisms like improvement of osmotic adjustment, minimizing Na^+^ uptake by roots and/or increasing Na^+^ efflux back to the soil, intracellular Na^+^ sequestration, potassium retention in the cytosol, tissue-specific Na^+^ sequestration, control of ion loading in xylem and oxidative stress tolerance (Yadav et al., 2012; Shabala, 2013; Mostofa et al., 2015).

The contrasting geographical position accompanied by monsoon climate, low and almost flat topography and range of rivers makes Bangladesh highly vulnerable to natural disasters, predominantly in coastal regions that accounts for 10% of the landmasses (Abdullah and Rahman, 2015). In many areas of southwest, south central, and southeast zone of coastal belt of Bangladesh, salinization has arisen as a serious problem, affecting the coastal ecosystems by degrading soil fertility, freshwater bodies and regeneration capacity of mangroves (Soil Resources Development Institute (SRDI), 2010). There are assorted species of halophytes being suitable to grow in different saline regions throughout the world, including Bangladesh. One of them is *Acacia auriculiformis* (acacia) that is native to Papua New Guinea, Northern Australia and Indonesia, which was brought for cultivation in Bangladesh in 1980s due to its fast growing nature, ensuring an adequate wood supply to sustain the country’s existing wood-based industries (Islam et al., 2013). It has been reported that *A. auriculiformis* is one of the most popular species in social forestry and cropland agroforestry, which has the potential to grow and survive in a wide range of territories and environments, and is well acclimatized to salty environments (Chowdhury et al., 2009). Thus, introduction of *A. auriculiformis*, as a salt-tolerant species, could be an important strategy in conserving ecology and wood production in the salt-affected regions of Bangladesh (Abdullah et al., 2015). However, the salt-tolerant limit and the defense mechanisms underlying salt-tolerant capacity in *A. auriculiformis* are still elusive. Moreover, no study has been conducted at morpho-physiological, biochemical and cellular levels to understand the mechanisms associated with the adaptability of *A. auriculiformis* under salt stress.

Therefore, in the present study, we aimed to examine the effects of a gradient of salinity on the growth and development of *A. auriculiformis*. At the same time, we have taken morphological, physiological, biochemical and anatomical approaches to get an insight into the salt tolerance mechanisms of *A. auriculiformis* by assessing various parameters, including (i) plant growth and biomass, (ii) salt uptake and accumulation, (iii) mineral homeostasis, (iv) photosynthetic pigments (e.g. chlorophylls), (v) stomatal conductance, (vi) lipid peroxidation, (vii) osmoregulation, (viii) anatomical features of roots, stems and phyllodes, and (ix) salt exclusion and tissue tolerance index in response to increasing doses of external salinity.

## Materials and Methods

### Study Location, Plant Materials, and Growth Conditions

A pot experiment was designed at the research field of the Department of Agroforestry and Environment, Bangabandhu Sheikh Mujibur Rahman Agricultural University, Bangladesh (24° 09′ N; 90° 26′ E) for a period of 5 months (January 2014– June 2014). The study site is 8.2 m above the sea level and characterized by a sub-tropical climate with hot summer and mild winter. The minimum and maximum temperatures of the research area were fluctuated between 7–23°C and 28–39°C, respectively, during the experimental period. One-year-old *A. auriculiformis* seedlings of uniform size were collected from Bangladesh Agricultural Development Corporation, Gazipur, Bangladesh, and each seedling was transplanted into each pot (26.5 cm in height and 27.5 cm in diameter). The pots were previously filled with soil mixture that was prepared by mixing oven-dried soil and cow dung (4:1). Each pot contained 12.04 kg of soil mixture and the soil moisture content was approximately 17% of field capacity. The soil was also treated with formaldehyde to curtail the probability of developing soil-borne diseases. In addition to cow dung, 1 L of liquid fertilizer containing urea, triple super phosphate and potassium sulfate (3% of each component) was applied to each pot at 30-day interval during the experimental period.

### Treatments and Experimental Design

After transplanting the seedlings into pots, the plants were allowed to grow for 10 days for adaptation prior to being treated with salt stress. Seawater collected from southern coastal area (Cox’s Bazar sea beach) of Bangladesh (electrical conductivity of 49.6 dS m^-1^, and Na^+^, K^+^, Ca^2+^ and Mg^2+^ concentrations of 478.70, 10.38, 9.90 and 56.08 mmol L^-1^, respectively) was diluted with tap water to formulate 4, 8 and 12 dS m^-1^ of salinity. For imposing salt stress, the uniformly grown plants were irrigated with the diluted seawater (300 mL) at 3-day interval for a period of 150 days. The control plants were irrigated with tap water devoid of seawater (0 dS m^-1^). Root, stem and phyllode samples were harvested at days 90^th^ and 150^th^ of salt treatments for determining various physiological and biochemical parameters. The experiment was conducted in randomized complete block design with five replicates in each treatment, and each replicate comprised one plant.

### Determination of Growth Parameters

Plant height and phyllode area were measured at days 90^th^ and 150^th^ of salt treatments using measuring scale and Area Meter (AM 350, ADC BioScientific Ltd), respectively. For determining biomasses of phyllodes and roots, plants were carefully uprooted at day 150^th^ of salt imposition and washed with distilled water to remove adherent soil. Dry weights (DWs) of phyllodes and roots were measured following the method described by Mostofa et al. (2015).

### Estimating Ion Contents in Phyllodes and Roots

The oven-dried phyllode and root samples were ground into powder and kept in a desiccator for determining the contents of Na^+^, K^+^, Ca^2+^ and Mg^2+^. After desiccation, the powdered samples were used to determine the ion contents by an atomic absorption spectrophotometer (Model No. 170-30. HITACHI, Japan) following the method of Roni et al. (2014).

### Determination of Phyllode Relative Water Content, Chlorophyll Contents, Stomatal Conductance and Stomata Number

The fresh, turgid and DWs of the phyllodes were used to determine phyllode relative water content (RWC) according to the formula developed by Zhang et al. (2014). Chlorophyll (Chl) contents of freshly collected phyllode samples were determined using 80% acetone as previously described by Misratia et al. (2013). Stomatal conductance of the adaxial part of the phyllodes was determined following the method of Pask and Pietragalla (2012) using a leaf porometer (SC-1, Decagon Devices, USA). To study the salinity-induced changes in stomatal distribution, phyllode epidermal peels were used for counting stomata number in the adaxial part by microscope (Olympus BX51, Tokyo, Japan).

### Contents of Proline, Total Free Amino Acids and Soluble Proteins

The levels of proline (Pro), total free amino acids and soluble proteins in phyllodes were determined following the methods described by Lowry et al. (1951), Lee and Takahashi (1966), and Bates et al. (1973), respectively.

### Electrolyte Leakage and Lipid Peroxidation

Electrolyte leakage (EL) and the content of lipid peroxidation product [malondialdehyde (MDA)] in phyllodes were determined according to Lutts et al. (1996) and Tayebimeigooni et al. (2012), respectively.

### Contents of Total Soluble Sugars, Reducing Sugars and Total Carbohydrates

The contents of total soluble sugars and reducing sugars, and total carbohydrates in phyllode samples were colorimetrically estimated according to the method of Somogyi (1952) and DuBois et al. (1956), respectively.

### Anatomical Features of Phyllode, Stem, and Root Tissues

Salinity-induced anatomical changes were examined by sectioning phyllode, stem, and root tissues under the light microscope (Leica Microsystems Ltd., version 3.0.0, Switzerland) using a cleaned razor blade (Gillette, Germany). The sections from control and seawater-treated plants were stained separately with safranin and fast-green following the method described by Sakai (1973). All sections were observed under bright- and dark-field compound microscope (Olympus BX51, Tokyo, Japan).

### Salt Tolerance Index, Tissue Tolerance Index and Na^+^ Exclusion

Salt tolerance index (STI) was calculated by using the following formula developed by Seydi (2003):

$$\mathrm{STI}\;=\frac{\mathrm{TDW}\;\mathrm{at}\;\mathrm{Sx}}{\mathrm{TDW}\;\mathrm{at}\;\mathrm{SI}}\times \;100$$

where, TDW = total DW, SI = control treatment, Sx = salt treatment.

Tissue tolerance was evaluated using the formula below according to Rajendran et al. (2009), with a slight modification:

$$\mathrm{Tissue}\;\mathrm{tolerance}=\frac{\left(\mathrm{Total}\;\mathrm{phyllode}\;\mathrm{area}-\mathrm{Predicted}\;\mathrm{salt-induced}\;\mathrm{senescene}\right)}{\mathrm{Total}\;\mathrm{phyllode}\;\mathrm{area}}\times \;4^{\mathrm{th}}\;\mathrm{phyllode}\;[{\mathrm{Na}}^{+}]$$

Total phyllode area was determined by summing up phyllode leaf area of total number of phyllodes of a plant, while senescenced area was determined by summing up total senescenced phyllode area. Projected phyllode area was calculated according to Keramatlou et al. (2015) with slight modification by multiple regression equation using leaf area as a dependent variable, which regressed with different independent variables, including length (L), width (W), L^2^, W^2^, (L+W)^2^ and the product L × W. The final model was selected based on the combination (Supplementary Figure 1) of the highest coefficient of determination (*r*^2^). The predicted area of natural senescence in salt-treated plants was calculated using the standard equation developed by regression analysis between projected phyllode area and senescenced phyllode area (Supplementary Figure 2).

$$\mathrm{Tissue}\;\mathrm{tolerance}=\frac{\left(\mathrm{Total}\;\mathrm{phyllode}\;\mathrm{area}-\mathrm{Predicted}\;\mathrm{salt-induced}\;\mathrm{senescene}\right)}{\mathrm{Total}\;\mathrm{phyllode}\;\mathrm{area}}\times \;4^{\mathrm{th}}\;\mathrm{phyllode}\;[{\mathrm{Na}}^{+}]$$

The predicted salt-induced senescenced area was estimated according to the equation as follows:

Predicted natural senescence = (Total phyllode area ×​​​ 0.0042) − 1090.6

The tissue tolerance index (TTI) was calculated by dividing the value of tissue tolerance at different doses by the value of the highest tissue tolerance obtained at any dose of salinity.

Na^+^ exclusion was determined as the ratio of the Na^+^ concentration in the 4^th^ phyllode and the Na^+^ concentration in the soil sample using the equation described by Munns (2005) with a slight modification as below:

Predicted salt-induced senescence = Salt-induced senescence area - predicted natural senescence

### Statistical Analysis

The data were subjected to a one-way or two-way analysis of variance (ANOVA), and different letters indicate significant differences among treatments at *P* < 0.05, according to least significant difference (LSD) test using Statistix 10 package. Data presented are means ± standard errors (SEs) of five replications (*n* = 5) for each treatment.

## Results

### Effects of Salinity on Growth, Biomass and Stomatal Density of *A. auriculiformis*

Salinity had considerable negative effects on the growth of *A. auriculiformis*, and the overall growth performance gradually declined upon increasing the level of salt stress for a period of 150 days (**Tables 1**, **2**). Salt stress caused a significant reduction of plant height by 15, 25 and 31% at day 90^th^, and 21, 30 and 39% at day 150^th^ after exposure of plants to 4, 8 and 12 dS m^-1^ salinity levels, respectively, as compared with the respective control values (**Table 1**). Reduction of phyllode area progressively augmented with increasing salinity levels, and phyllode area was found to be reduced by 24, 44 and 54% at day 90^th^, and 39, 56 and 68% at day 150^th^ in comparison with control after application of 4, 8 and 12 dS m^-1^ salinity levels to plants, respectively (**Table 1**).

**Table 1:** Effects of salinity on plant height, phyllode area, and the levels of chlorophyll (Chl) *a*, Chl *b* and total Chls, and Chl *a*/Chl *b* ratio in *Acacia auriculiformis* exposed to different levels of salinity at days 90^th^ and 150^th^ after salt treatment.

Duration of treatment (day)	Salinity levels (dS m^-1^)	Plant height (cm)	Phyllode area (× 10^4^ mm^2^)	Chl *a* (mg g^-1^ FW)	Chl *b* (mg g^-1^ FW)	Total Chls (mg g^-1^ FW)	Chl *a*/Chl *b* ratio
90	0	86.80 ± 0.47^c^	67.57 ± 20.42^e^	6.72 ± 0.06^a^	2.61 ± 0.03^a^	9.34 ± 0.08^a^	2.57 ± 0.02^d^
	4	74.16 ± 1.49^d^	51.65 ± 9.65^f^	5.56 ± 0.04^b^	2.38 ± 0.02^b^	8.06 ± 0.04^b^	2.37 ± 0.02^e^
	8	65.08 ± 0.26^e^	38.10 ± 7.87^g^	5.20 ± 0.03^c^	2.29 ± 0.05^b^	7.50 ± 0.10^c^	2.26 ± 0.03^f^
	12	59.89 ± 0.79^f^	30.81 ± 3.40^g^	4.03 ± 0.05^e^	1.98 ± 0.03^c^	6.00 ± 0.04^d^	2.03 ± 0.03^g^
150	0	129.06 ± 4.21^a^	297 ± 44.12^a^	4.80 ± 0.07^d^	1.25 ± 0.02^d^	6.05 ± 0.09^d^	3.83 ± 0.03^a^
	4	102.4 ± 1.05^b^	182 ± 23.08^b^	3.92 ± 0.04^e^	1.06 ± 0.01^e^	4.98 ± 0.06^e^	3.69 ± 0.005^b^
	8	89.90 ± 1.55^c^	130 ± 19.95^c^	3.43 ± 0.03^f^	0.95 ± 0.01^f^	4.38 ± 0.05^f^	3.61 ± 0.02^b^
	12	78.20 ± 1.37^d^	93.72 ± 18.43^d^	2.80 ± 0.05^g^	0.81 ± 0.01^g^	3.61 ± 0.06^g^	3.45 ± 0.02^c^

*Values are means ± standard errors of five independent replications (*n* = 5). Different superscripted letters within the column indicate statistically significant differences among the treatments according to a least significant difference test (*P* < 0.05). FW, fresh weight*.

The negative consequences of salinity on growth performance of *A. auriculiformis* were also assessed by determining DW of phyllodes and roots, DW-based phyllode/root ratio and root length after 150 days of salt treatment (**Table 2**). DW of salt-stressed phyllodes reduced by 26, 38 and 49%, while that of salt-treated roots by 31, 43 and 54% at 4, 8 and 12 dS m^-1^ salinity levels, respectively, compared with that of control. Consequently, DW-based phyllode/root ratio increased by 8, 8 and 12% at 4, 8 and 12 dS m^-1^ saline levels, respectively, in comparison with control. Careful evaluation of the length of roots showed that root length decreased by 22, 32 and 43% at 4, 8 and 12 dS m^-1^ saline levels, respectively, when compared with control (**Table 2**). STI, a reliable criterion for salt tolerance (Abbas et al., 2013), was also differentially affected by salt stress. The obtained results showed that STI dramatically attenuated as the level of salinity increased (**Table 2**). Nevertheless, STI decreased only by 31% despite that the level of salinity used was increased three times from 4 to 12 dS m^-1^. Seawater-induced salinity significantly affected the stomatal density in the adaxial part of the 4^th^ phyllode and the number of stomata was proportionally declined with the levels of salinity (**Table 2**). Specifically, the stomata density in the phyllode of salt-stressed *A. auriculiformis* was decreased by 15, 27 and 47% at 4, 8 and 12 dS m^-1^ salinity levels, respectively, in comparison with that of salt-free control plants.

**Table 2:** Effects of seawater-induced salinity on dry weight (DW) of phyllodes and roots, DW-based phyllode/root ratio, root length, salt tolerance index (STI) and stomatal density of *A. auriculiformis* at day 150^th^ of salt treatment.

Salinity levels (dS m^-1^)	Dry biomass (g)		DW-based phyllode/root ratio	Root length (cm)	STI	Stomatal density (stomata mm^-2^)
	Phyllodes	Roots				
0	328.21 ± 4.21^a^	32.56 ± 0.36^a^	10.08 ± 0.12^a^	110.28 ± 0.41^a^	–	60 ± 1.16^a^
4	243.69 ± 2.96^b^	22.42 ± 0.13^b^	10.86 ± 0.07^ab^	86.04 ± 0.97^b^	73.78 ± 0.83^a^	51 ± 0.46^b^
8	201.93 ± 2.49^c^	18.47 ± 0.16^c^	10.93 ± 0.13^b^	74.50 ± 0.45^c^	61.15 ± 1.40^b^	44 ± 0.85^c^
12	168.96 ± 1.29^d^	14.96 ± 0.19^d^	11.29 ± 0.12^c^	62.82 ± 1.25^d^	50.89 ± 0.67^c^	32 ± 1.12^d^

*Values are means ± standard errors of five independent replications (*n* = 5). Different superscripted letters within the column indicate statistically significant differences among the treatments according to a least significant difference test (*P* < 0.05)*.

### Effects of Salinity on Phyllode Water Status and Stomatal Conductance

**FIGURE 1:**
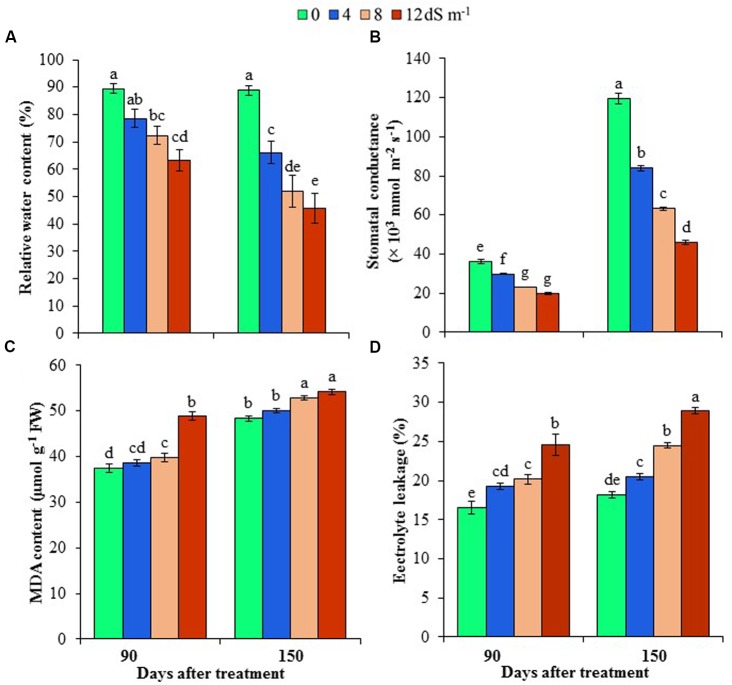
**Effects of salinity on (A)** relative water content, **(B)** stomatal conductance, **(C)** malondialdehyde (MDA) content and **(D)** electrolyte leakage of *Acacia auriculiformis* at days 90^th^ and 150^th^ of exposure to different levels of salinity (0, 4, 8 and 12 dS m^-1^). Bars represent standard errors of the means (*n* = 5). Different letters indicate significant differences among the treatments at *P* < 0.05, according to a least significant difference test. FW, fresh weight.

Phyllode RWC was determined at different stages of development of *A. auriculiformis* after salt treatment, and RWC was observed to reduce in a dose- and exposure time-dependent manner. In response to seawater-induced salinity, phyllode RWC decreased by 12, 19 and 29% at day 90^th^, and 26, 41 and 49% at day 150^th^ under 4, 8 and 12 dS m^-1^ salinity levels, respectively, compared with control (**Figure 1A**). The influence of salinity stress on stomatal conductance of *A. auriculiformis* plants (**Figure 1B**) was similar to that on RWC. Stomatal conductance decreased in a salt concentration-dependent manner by 18, 36 and 45% at day 90^th^, and 30, 47 and 62% at day 150^th^ of imposition of 4, 8 and 12 dS m^-1^ salinity, respectively, as compared with control. Our data also indicated that the stomatal conductance of the old plants were significantly higher than that of young plants under both normal and salt stress conditions.

### Effects of Salinity on Chlorophyll Contents and Ratio

Irrigation of *A. auriculiformis* plants with diluted seawater caused gradual and significant decreases in the contents of Chl *a* (by 17, 23 and 40% at day 90^th^, and 18, 29 and 42% at day 150^th^), Chl *b* (by 9, 12 and 24% at day 90^th^, and 15, 24 and 35% at day 150^th^) and total Chls (by 14, 20 and 36% at day 90^th^, and 18, 28 and 40% at day 150^th^) following imposition of 4, 8 and 12 dS m^-1^ salinity, respectively, compared with plants irrigated with tap water (**Table 1**). Severe salt stress (12 dS m^-1^) exhibited a higher reduction in Chl *a* content (by 42%) than in Chl *b* (by 35%), leading to a conspicuous decrease in the overall Chl content (40%), when compared with control at day 150^th^ of salt treatment. Additionally, the decreasing patterns of Chl *a* and Chl *b* also significantly altered Chl *a*/Chl *b* ratio in plants treated with diluted seawater (**Table 1**).

### Effects of Salinity on Cell Membrane Stability

To assess the cell membrane integrity under salt stress, we determined the levels of lipid peroxidation product MDA and EL in the phyllodes of *A. auriculiformis* (**Figures 1C,D**). There were no significant changes in MDA contents at low salinity; whereas at moderate and high salinity (8 and 12 dS m^-1^), MDA content significantly increased by 6 and 30% at day 90^th^ of salt treatment compared with control. On the other hand, at day 150^th^ of salt imposition, MDA content significantly increased by 9 and 12% at moderate (8 dS m^-1^) and high (12 dS m^-1^) salinity levels, respectively, relative to control. There was also a sharp increase in the content of EL upon increasing the levels of salinity. Nevertheless, the increment of EL levels showed the maximum at the highest salinity dose (12 dS m^-1^), with 49 and 59% higher EL at days 90^th^ and 150^th^, respectively, than that of untreated respective control.

### Effects of Salinity on the Levels of Proline, Total Free Amino Acids, Total Soluble Sugars, Reducing Sugars, Total Carbohydrates and Soluble Proteins

**FIGURE 2:**
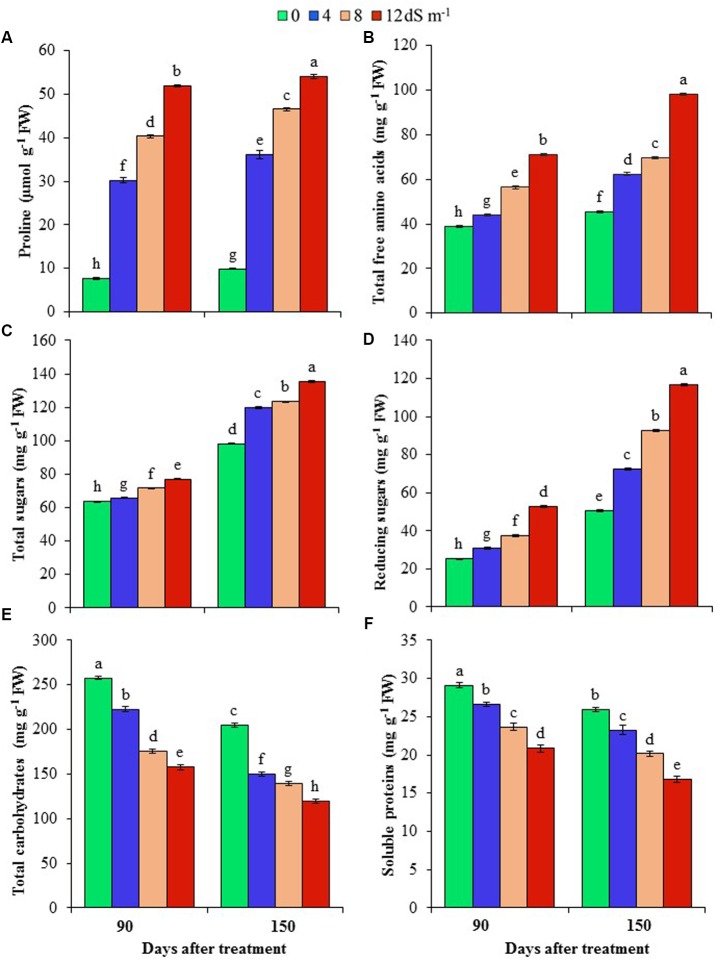
**Effects of salinity on (A)** proline, **(B)** total free amino acids, **(C)** total sugars, **(D)** reducing sugars, **(E)** total carbohydrates and **(F)** soluble proteins of *A. auriculiformis* at days 90^th^ and 150^th^ of exposure to different levels of salinity (0, 4, 8 and 12 dS m^-1^). Bars represent standard errors of the means (*n* = 5). Different letters indicate significant differences among the treatments at *P* < 0.05, according to a least significant difference test. FW, fresh weight.

To understand the mechanisms associated with osmotic adjustment under salt stress, we have determined the levels of Pro, total free amino acids, total sugars, total carbohydrates, reducing sugars, and soluble proteins in *A. auriculiformis* plants under normal and salt stress conditions. Pro content remarkably increased by 298, 430 and 583% at day 90^th^, and 265, 371 and 447% at day 150^th^ after imposition of 4, 8 and 12 dS m^-1^ diluted seawater, respectively, compared with control (**Figure 2A**). There was a gradual increase in the content of total free amino acids upon increasing the levels of salinity and exposure times (**Figure 2B**). At day 150^th^ of salt treatment, the levels of total free amino acids were elevated by 38, 54 and 116% at 4, 8, and 12 dS m^-1^ salinity levels, respectively, in comparison with control. Similar to the trend of total free amino acid contents, there was a positive relationship between salt concentrations and the levels of total soluble sugars and reducing sugars, and the maximum levels were noted at the highest level of salinity (12 dS m^-1^) at both days 90^th^ and 150^th^ of plant exposure to salt stress. At day 90^th^ of salt stress, the level of total soluble sugars was enhanced by 4, 13 and 21% (**Figure 2C**), and that of reducing sugars by 22, 48 and 109% (**Figure 2D**) at 4, 8 and 12 dS m^-1^ salinity levels, respectively, compared with control. Upon increasing the salt-exposure time for an additional 60 days, the level of total sugars further increased by 22, 26 and 38%, and that of reducing sugars by 44, 84 and 131% at 4, 8 and 12 dS m^-1^ salinity levels, respectively, as compared with control (**Figures 2C,D**). On the other hand, the contents of total carbohydrates and soluble proteins were negatively correlated with increasing doses of seawater (**Figures 2E,F**). At day 90^th^ of salt treatment, total carbohydrate content decreased by 14, 32 and 39%, while that of soluble proteins by 9, 19 and 28% at 4, 8 and 12 dS m^-1^ salinity levels, respectively, compared with control (**Figures 2E,F**). The contents of these biomolecules were further declined at day 150^th^ of salt exposure, and their values reached the lowest levels (42 and 35% for total carbohydrates and soluble proteins, respectively) at the highest concentration of salinity (12 dS m^-1^) (**Figures 2E,F**).

### Effects of Salinity on Ion Homeostases

**FIGURE 3:**
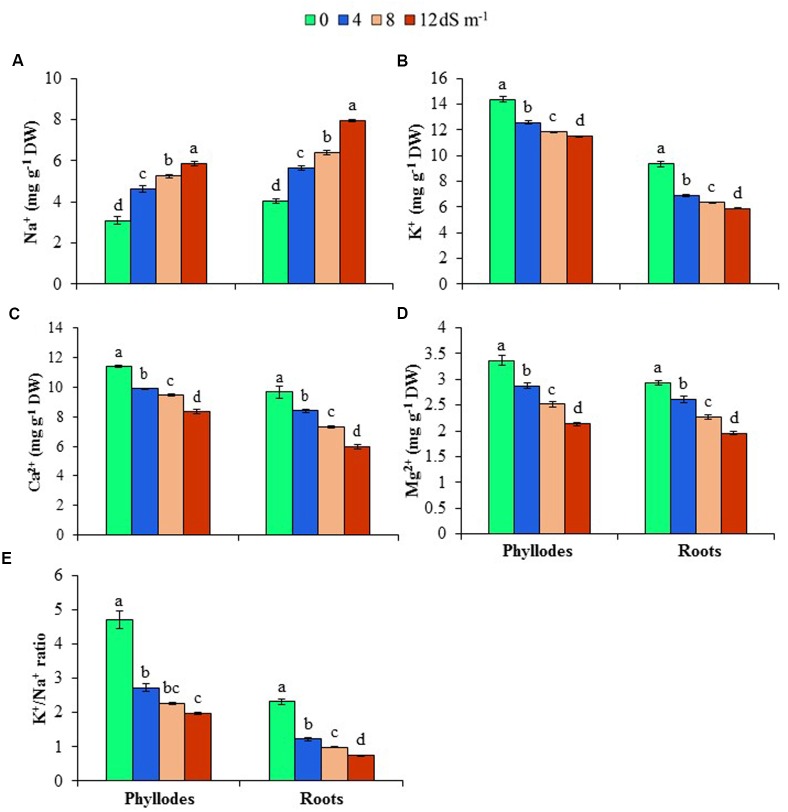
**Effects of salinity on (A)** Na^+^, **(B)** K^+^, **(C)** Ca^2+^, **(D)** Mg^2+^ and **(E)** K^+^/Na^+^ ratio at day 150^th^ of exposure of *A. auriculiformis* plants to different levels of salinity (0, 4, 8 and 12 dS m^-1^). Bars represent standard errors of the means (*n* = 5). Different letters indicate significant differences among the treatments at *P* < 0.05, according to a least significant difference test. DW, dry weight.

To get an insight into ion homeostasis mechanism of *A. auriculiformis*, we measured the contents of Na^+^, K^+^, Ca^2+^ and Mg^2+^ in roots and phyllodes at day 150^th^ of salt treatment. As shown in **Figures 3A,B**, treatment of plants with 4, 8 and 12 dS m^-1^ salinity levels resulted in a noticeable increase in the content of Na^+^ (39, 58 and 96% in roots, and 50, 70 and 89% in phyllodes, respectively), whereas a significant reduction in K^+^ content (26, 32 and 37% in roots, and 13, 18 and 20% in phyllodes, respectively) in salt-stressed plants compared with untreated control plants. It is worth mentioning that lower levels of Na^+^ and higher levels of K^+^ were accumulated in phyllodes, when compared with those in roots, indicating a higher salt tolerance tendency of *A. auriculiformis.* We also noted a significant decrease in the contents of Ca^2+^ and Mg^2+^ in both roots and phyllodes of salt-stressed plants, as compared with that in seawater-free control plants (**Figures 3C,D**). The levels of Ca^2+^ and Mg^2+^ were also considerably higher in phyllodes than in roots of salt-stressed plants. We also calculated the K^+^/Na^+^ ratios, and found that salinity caused a decreasing trend of the ratios in both phyllodes and roots with progressive increase of salinity levels. However, it is worth mentioning that phyllodes displayed higher K^+^/Na^+^ ratios than roots (**Figure 3E**). Thus, retention of Na^+^ in roots and maintenance of higher levels of K^+^, Ca^2+^ and Mg^2+^ in phyllodes enabled *A. auriculiformis* to adjust its ion homeostasis under salt stress.

### Effects of Salinity on Anatomical Features of Phyllodes, Stems and Roots

**FIGURE 4:**
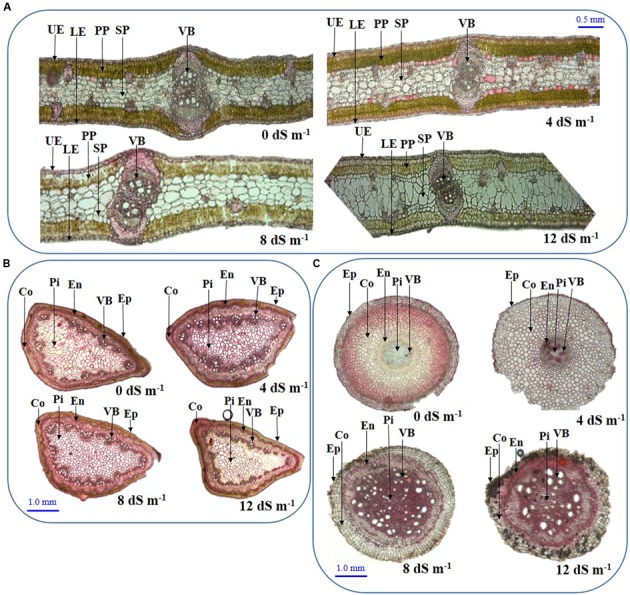
**Effects of salinity on the anatomical features of (A)** phyllode, **(B)** stem and **(C)** root at day 150^th^ of exposure of *A. auriculiformis* plants treated with various levels of salinity (0, 4, 8 and 12 dS m^-1^). UE, upper epidermes; PP, palisade parenchyma; SP, spongy parenchyma; VB, vascular bundle; LE, lower epidermis; Co, cortex; En, endodermis; Ep, epidermis; Pi, pith.

To assess the anatomical adaptation of *A. auriculiformis* during salt stress, we analyzed the cross sections of phyllodes, stems and roots at day 150^th^ of plant exposure to different levels of salinity (**Figures 4A–C**). Our observations implied that the anatomical features of phyllodes, stems and roots significantly altered when *A. auriculiformis* plants were exposed to external salinity. In phyllodes, the thickness of upper and lower epidermises increased at low and moderate salinity levels (8 and 12 dS m^-1^) but decreased at high salinity (12 dS m^-1^), relative to control counterparts (**Figure 4A**). Progressive increment of salinity levels triggered the loss of Chl substances in palisade parenchyma tissues. We also observed that the size of the spongy parenchymal layer gradually increased with increasing salinity levels. Salt stress resulted in a significant decrease in cortical area of stems of *A. auriculiformis* but no significant variation was found in case of epidermis even at the highest dose of salinity (12 dS m^-1^) (**Figure 4B**). Endodermal thickness of stems was found to be increased with increasing salinity when compared with those of the stems of control plants (**Figure 4B**). The vascular bundles of the stems enlarged at low (4 dS m^-1^) and moderate salinity (8 dS m^-1^); but decreases at high salinity level (12 dS m^-1^). On the other hand, salt-mediated toxicity increased the number of the cells in pith area of stems up to 8 dS m^-1^ salinity; thereafter, a decrease in number of cells was observed at 12 dS m^-1^. Results in **Figure 4C** indicated that the thicknesses of endodermis, pith area and the diameter of vascular bundles increased upon increasing the salinity levels (**Figure 4C**). Interestingly, the cortical area in roots expanded at low intensity of salinity (4 dS m^-1^), then decreased at moderate and high levels of salinities (8 and 12 dS m^-1^). Moreover, salt stress triggered the increment of intercellular space and rupture of cell membrane of root tissues (**Figure 4C**), which might be resulted from the formation of aerenchyma, a unique adaption feature of salt-affected tissues (Flowers and Colmer, 2008).

### Sodium Exclusion and TTI

At day 150^th^ of plant exposure to different doses of salinity, the final concentration of Na^+^ in the fully expanded fourth phyllode of *A. auriculiformis* was considered for measuring the Na^+^ exclusion index (**Table 3**). The lowest and highest excluders of Na^+^ were given a value of 1 and 0, respectively, to develop a standardized Na^+^ exclusion index. According to this scale, exposure of plants to 4, 8 and 12 dS m^-1^ salinity exhibited a Na^+^ exclusion index of 0.91, 0.84 and 0.79, respectively, compared with control (1.0). The data presented in **Table 4** exemplified that plants with low degree of salt-induced senescence and high Na^+^ concentrations were deemed to be tissue-tolerant. A measure for tissue tolerance, therefore, needs to consider the amount of Na^+^ accumulated in the phyllodes. Before calculating the TTI, it was necessary to evaluate natural phyllode senescence and necrosis in salt-stressed plants in order to determine the extent of Na^+^ prompted senescence. The index was scaled to a range between 0 and 1 by dividing the tolerance value of those salinity levels by the highest tolerance value. Thus, the highest tissue tolerance would have a value of 1, while the theoretically lowest tissue tolerance would have a value of 0. Our data analysis indicated that *A. auriculiformis* had the highest TTI (1) at 4 dS m^-1^ salinity followed by 8 dS m^-1^ (0.91) and 12 dS m^-1^ (0.74) salinity levels.

**Table 3:** Effects of salinity on Na^+^ exclusion index of *A. auriculiformis* grown under different levels of salinity at day 150^th^ of salt treatment.

Salinity levels (dS m^-1^)	Level of Na^+^ in 4^th^ phyllode (mg g^-1^ DW)	Level of Na^+^ in soil (mg g^-1^ DW)	Na^+^ exclusion (%)	Na^+^ exclusion index
0	3.08 ± 0.17^d^	11.78 ± 0.11^d^	99.74 ± 0.015^a^	1
4	4.63 ± 0.14^c^	16.28 ± 0.13^c^	99.71 ± 0.008^ab^	0.91
8	5.25 ± 0.07^b^	17.12 ± 0.06^b^	99.69 ± 0.005^bc^	0.84
12	5.84 ± 0.11^a^	17.96 ± 0.04^a^	99.67 ± 0.006^c^	0.79

*Values are means ± standard errors of five independent replications (*n* = 5). Different superscripted letters within the column indicate statistically significant differences among the treatments according to a least significant difference test (*P* < 0.05). DW, dry weight*.

**Table 4:** Effects of salinity on tissue tolerance index (TTI) of *A. auriculiformis* grown under different levels of salinity at day 150^th^ of salt treatment.

Salinity levels (dS m^-1^)	Measured area (×10^4^ mm^2^)		Predicted senescenced area (×10^4^ mm^2^)		Fourth phyllode Na^+^ (mg g^-1^ DW)	Tissue tolerance	TTI
	Total phyllode area	Senescenced area	Natural area	Salt-induced area			
4	181.86 ± 2.30^a^	35.96 ± 0.54^c^	0.65 ± 0.01^a^	35.31 ± 0.55^c^	4.63 ± 0.14^c^	3.73 ± 0.14^a^	1
8	129.62 ± 1.99^b^	45.78 ± 0.24^b^	0.43 ± 0.01^b^	45.35 ± 0.24^b^	5.25 ± 0.07^b^	3.40 ± 0.06^b^	0.91
12	93.72 ± 1.84^c^	49.31 ± 0.36^a^	0.28 ± 0.009^c^	49.03 ± 0.36^a^	5.84 ± 0.11^a^	2.77 ± 0.18^c^	0.74

*Values are means ± standard errors of five independent replications (*n* = 5). Different superscripted letters within the column indicate statistically significant differences among the treatments according to a least significant difference test (*P* < 0.05). DW, dry weight*.

## Discussion

**FIGURE 5:**
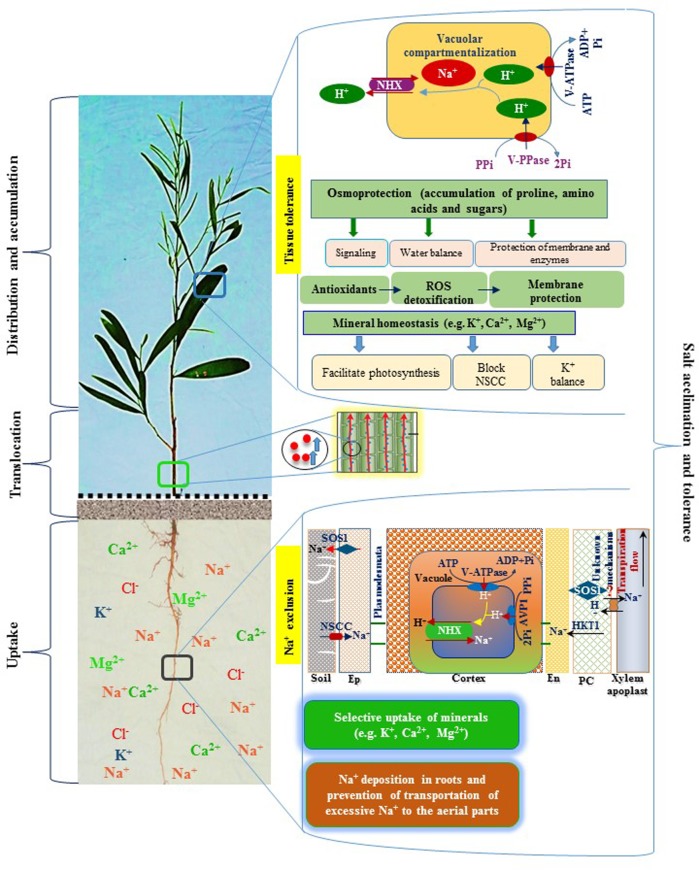
**A schematic diagram representing salt tolerance mechanism of *A. auriculiformis***. *A. auriculiformis* showed adaptation to salinity by employing various mechanisms, including Na^+^ exclusion and tissue tolerance. Salt exposure causes a greater accumulation of Na^+^ in the roots, thereby limiting transportation of excessive Na^+^ in the aerial parts of *A. auriculiformis.* The roots also showed ion selectivity over excessive Na^+^ and enhanced the levels of micronutrients like K^+^, Ca^2+^ and Mg^2+^ in the phyllodes. The roots may also adopt Na^+^ exclusion mechanism by preventing accumulation of toxic concentrations of Na^+^ within phyllodes, mainly by (i) retrieving Na^+^ from the xylem, (ii) compartmenting Na^+^ in vacuoles of cortical cells, and (iii) effluxing of Na^+^ back to the soil (Roy et al., 2014). Once Na^+^ is accumulated in the leaves, plants enhance tissue tolerance by seizing Na^+^ in the vacuoles, a process involving ion transporters and proton pumps. Tissue tolerance also attributes to the accumulation of osmoprotective compounds, such as proline, amino acids and sugars, which are involved in stress signaling, water balance and protection of membranes and enzymes. Salt stress induces antioxidant systems to fight against reactive oxygen species (ROS), leading to the reduction of ROS-mediated membrane damage. Salt-affected tissues also maintain mineral homeostasis, which contributes to the photosynthesis, inhibition of non-selective cation channels (NSCC) and enhancement of K^+^ levels. Collectively, Na^+^ exclusion, tissue tolerance, osmoprotection and mineral homeostasis play vital roles in salt acclimation and tolerance in *A. auriculiformis*. Ep, epidermis; En, endodermis; PC, parenchyma.

One of the general consequences of salt toxicity is to inhibit plant growth, resulting in the generation of stunted plants. At above the threshold level of salt, the growth rate and biomass of all plants progressively decrease, although the degree of damage varies among plant species (Parida et al., 2016). In the current study, plant growth-related parameters, such as plant height, phyllode area, root length, and root and phyllode DWs, were significantly affected upon increasing the levels of salt, indicating salinity-induced reduction of the growth performance of *A. auriculiformis* by interfering various parameters upon which its growth relies (**Tables 1**, **2**). We observed a salt-induced increasing trend of phyllode/root ratio (**Table 2**), which was supported by the findings of Ramoliya et al. (2004). The high phyllode/root biomass ratio in salt-exposed plants suggests that acacia plants may adopt a strategy, which is functionally associated with the need of salt-stressed plants to minimize the uptake of toxic ions to the phyllodes, while maintaining better growth response under salinity (Theerawitaya et al., 2015; **Figure 5**).

Photosynthetic efficiency determines the overall performance of plants, which is reflected by growth and biomass parameters. Under salt stress, phyllode Chl content could be altered due to impaired biosynthesis and accelerated degradation of the pigments (Neelam and Subramanyam, 2013). Therefore, the levels of photosynthetic pigments, such as Chl *a* and Chl *b*, are vital for steady photosynthesis in plants during salt stress. It has been reported that photosynthesis in some halophytes remains unaffected by salinity or even increases at low salinity (Flowers and Colmer, 2015). In the current study, the levels of Chl *a* and *b*, as well as total Chls, were progressively declined with the rise of saline level and exposure time, indicating that the photosynthetic performance of *A. auriculiformis* was hampered, which contributed to the decrease in plant biomass (**Tables 1**, **2**). The reduction in Chl levels and consequent biomass loss were evident in other studies under salt stress, and the possible cause was attributed to the direct effects of accumulated Na^+^ and ROS (Qin et al., 2010; Mostofa et al., 2015). In accordance with other reports, our results also implied that Chl *a* is more sensitive to salinity than Chl *b* (Li et al., 2008; Peng et al., 2016). Chl *a* is the most abundant and integral part of the light-harvesting complex, whereas Chl *b* as an accessory pigment acts indirectly in photosynthesis by transmitting photons to the Chl *a* (Chen, 2014; Gururani et al., 2015). Thus, a higher level of Chl *a* than Chl *b* at all concentrations of seawater might have contributed to the improved efficiency of *A. auriculiformis* in resisting salt-induced loss of photosynthetic capacity (**Table 1**), as was repoted in *Atriplex helimus* by Bendaly et al. (2016)

Salt-induced decline in photosynthetic pigments in *A. auriculiformis* was coincided with decreases in RWC, stomatal density and conductance, and membrane degradation (**Tables 1**, **2** and **Figures 1A–D**). In this study, we observed an incremental reduction of phyllode RWC with increasing the concentration and exposure period of seawater, indicating salt-mediated induction of physiological water deficit in *A. auriculiformis* (**Figure 1A**). Decreases in stomata number and conductance in phyllodes might be an adaptation mechanism that *A. auriculiformis* employs to optimize water use efficiency and to make spaces for sequestrating more Na^+^ under salt stress, as the similar tendency was also observed in other halophytes, such as *Chenopodium quinoa* (Shabala et al., 2012; Shabala, 2013). On the other hand, higher rates of stomatal conductance in mature leaves of old plants than that of young plants (**Figure 1B**) might be attributed to disparity of total stomatal number, stomatal morphology and number of chloroplasts, which is commonly found in the fully expanded leaves (Samarappuli, 1988; Xie and Luo, 2003). Fontana et al. (2017) demonstrated that increase in leaf area in mature leaves was associated with the increased stomatal density and conductance in the adaxial part, which contributed to higher water loss from older leaves through greater exchange of water vapor than that from younger leaves. In agreement with this finding, older acacia phyllodes lost more water compared with younger phyllodes under salt stress, indicating, at least partly, higher stomatal conductance of older phyllodes might contribute to the increased water loss rate (**Figures 1A,B**).

In addition, once it is accumulated at higher level in the tissues, Na^+^ initiates cellular toxicity by targeting cell membrane through the production of ROS. ROS mainly attacks the membrane, causing lipid peroxidation that leads to the generation of MDA and loss of essential electrolytes (Penella et al., 2016). MDA content and EL in acacia phyllodes increased to a lesser extent at low and moderate salinities (4–8 dS m^-1^); however, these two determinants drastically increased at severe salinity (12 dS m^-1^) and long-exposure period (150 days), indicating the capability of *A. auriculiformis* in resisting salt-induced membrane damage up to a certain level of salinity (**Figures 1C,D**). These data also demonstrated the correlation between the significant increase of MDA content and EL, and the growth inhibition and metabolic disturbance under severe salt stress (**Figures 1C,D** and **Table 2**), as previously reported in other species (Rangani et al., 2016).

Under osmotic stress, halophyte plants have been reported to accumulate low-molecular-weight compatible solutes and osmoprotectants in sub-cellular compartments to reduce water loss (Flowers and Colmer, 2015). We observed that *A. auriculiformis* significantly enhanced the accumulation of Pro, total free amino acids and total soluble sugars in leaves that might help salt-stressed plants adjust to water deficit conditions (**Figure 5**). Pro is known to provide improved protection against salinity by scavenging free radicals, stabilizing membranes, proteins and enzymes, and maintaining ion homeostasis (Kishor and Sreenivasulu, 2014). Salt-induced production of Pro has been demonstrated in both halophytes and glycophytes; however, halophytes accumulate more Pro under control and stressed conditions (Shiri et al., 2016). Additionally, by maintaining enhanced levels of free amino acids, halophytes are capable of satisfying the increased demand for amino acids during protein metabolism under salt stress. Moreover, elevated sugar levels in phyllodes indicated that *A. auriculiformis* responded positively in mitigating reduced RWC resulted from the increase in salt toxicity (**Figures 2A–D**, **5**). Interestingly, the observed declines in total carbohydrate and protein contents endorsed the increased levels of sugars and amino acids (**Figures 2B–F**), as well as enhanced rate of respiration to produce much ATP required for the activity of stressed cells (Khalafallah et al., 2013). In accordance with the previous findings (Slama et al., 2015; Parida et al., 2016), our results also support the notion that elevated levels of sugars due to increased degradation of carbohydrates by hydrolytic enzymes contribute to the osmotic protection under high salinity. However, the reduced levels of carbohydrates and proteins might also be resulted from the greater degradation and reduced synthesis of these biomolecules in response to salt stress, as was evidenced in another report (Rajaravindran and Natarajan, 2012).

The salt tolerance in plants is invariably associated with three major components, including (i) Na^+^ exclusion, (ii) Na^+^ extrusion and (iii) sequestration of Na^+^ in the vacuoles (**Figure 5**). Additionally, impact of salt stress on the uptake and transport of other minerals is also considered important determinant of salt tolerance mechanism in plants (Taïbi et al., 2016). In the present study, Na^+^ levels in the roots and phyllodes of *A. auriculiformis* were significantly increased, whereas K^+^ concentrations and the consequent K^+^/Na^+^ ratios were reduced in a concentration- and time-dependent manner (**Figures 3A,B,E**). These data indicate that competitive inhibition between Na^+^ and K^+^ absorptions resulted in a change in the balance of intracellular K^+^/Na^+^ ratio in acacia plants. However, Na^+^ concentrations in roots were significantly higher than that of phyllodes (**Figure 3A**), implying greater deposition of Na^+^ in the roots and reduced translocation of excessive Na^+^ from roots to aerial parts of acacia plants. This regulation of Na^+^ and K^+^ homeostases also implies the ability of *A. auriculiformis* to maintain a sufficient uptake of K^+^ in order to maintain a high cytosolic K^+^/Na^+^ ratio (**Figure 3E**), which is one of the key determinants of plant salt tolerance (Abideen et al., 2014). The significant higher levels of Ca^2+^ and Mg^2+^ in phyllodes than in roots of the seawater-stressed acacia plants (**Figures 3C,D**) also indicated that ion exclusion mechanisms reduced the antagonistic effects of Na^+^, hence accelerating the uptake of other beneficiary nutrients. K^+^ actively participates in activating enzymes, stabilizing protein synthesis, maintaining membrane potential and cytosolic pH, whereas Ca^2+^ plays pivotal roles in K^+^/Na^+^ selectivity and signal transduction, and Mg^2+^ regulates cellular pH and ionic balance in addition to its central role in pigment formation (Wu et al., 2013; Flowers et al., 2014; Song and Wang, 2015). Our results, therefore, suggest that the higher levels of K^+^, Ca^2+^ and Mg^2+^ in phyllodes than in roots sustain optimal operation of metabolic processes and overall performance of *A. auriculiformis* under salt stress (**Figures 3B–D**, **5**).

Salt stress also exerted severe load on the cellular organelles, and plants are accustomed to adjust the anatomical features in order to minimize the damage in presence of excessive NaCl. Phyllode anatomical observation showed increased thickness of upper and lower epidermises up to moderate salinity, indicating improved water use efficiency and sequestration of Na^+^ in salt-affected phyllodes (Shabala et al., 2012; Parida et al., 2016) (**Figure 4A**). However, reduced epidermal thickness at high salinity indicated limited cell division and growth response under extreme salinity. The decrease in vascular tissue area under excessive salinity suggested an adaptation strategy to reduce water loss, which is supported by the findings of Ristic and Cass (1991) and Kutlu et al. (2009). Reduction of vascular bundle size may also suggest reduced uptake of salt-water through xylem and minimization of water loss through transpiration.

The anatomy of stems was also severely altered upon imposing various doses of saline water (**Figure 4B**). The decrease in cortical area of stems might be a defensive strategy plants adapted to curtail growth under salinity by conserving essential energy for survival (Nawazish et al., 2006; Alam et al., 2015). Conversely, increased thickness of stem endodermis under salinity may be considered as a defensive strategy to reduce Na^+^ toxicity (**Figures 4B**, **5**).

Root is the primary organ to be affected by salinity and is known to remodel its anatomical features for adapting salt-induced adverse changes (Ji et al., 2013). The cross section of roots showed increased thickness of the endodermis and pith area (**Figure 4C**), thereby suggesting a reduced diffusion of Na^+^ to the roots (Parida et al., 2016). On the other hand, expanded sizes of vascular bundle of roots indicate that the salt-stressed plants tended to maintain better water status and nutrient levels for adjusting disturbed-metabolic processes (**Figure 4C**). Blackish spots and ruptured cells were also observed under elevated salinity (**Figure 4C**), which attributed to the formation of ROS and MDA (Gao et al., 2015). Additionally, the sizes of the cortical and pith areas of roots were found to be increased, which might enhance storage capacity to preserve more water to overcome unfavorable moisture conditions under salt stress.

**Figure 5** summarizes the potential mechanisms underlying salt tolerance of *A. auriculiformis*. Our findings conclude that exposure of plants to seawater-induced salinity imposed adverse effects on the performance of the whole plant, particularly by inhibiting growth and development. The higher STI at seedling stage indicate that the key mechanisms of salt tolerance in plants may be associated with (i) accumulation of compatible solutes like Pro, total sugars, reducing sugars and total free amino acids; (ii) increase amount of K^+^, Ca^2+^ and Mg^2+^ in phyllodes than roots; (iii) increase K^+^ retention in photosynthetic tissues through hindering Na^+^ uptake; (iv) anatomical adjustment by increasing the size of spongy parenchymal tissue of phyllodes, endodermal thickness of stems and roots, and pith area of roots; (v) efficient Na^+^ sequestration in vacuoles that would be facilitated by a decrease in stomatal density and (vi) the enhanced Na^+^ exclusion and TTI. Our findings support that *A. auriculiformis* is a suitable perennial tree species to be grown for conservation of soil and ecology, as well as earning income in salt-affected areas owing to its popular use as fuel wood and materials for making paper and furniture.

## Author Contributions

MMR, MAR, and SS conceived an designed the experiments. MMR, MAR, and SS conducted experiments. MMR and MGM analyzed and interpreted the data and results. MMR and MGM wrote and revised the manuscript. MGUM and MAK provided materials, monitored the experimental work and critically commented on the manuscript. All authors read and approved the final manuscript.

## Conflict of Interest Statement

The authors declare that the research was conducted in the absence of any commercial or financial relationships that could be construed as a potential conflict of interest.
