# Supplementary material for: Mechanistic Insight into Salt Tolerance of Acacia auriculiformis: The Importance of Ion Selectivity, Osmoprotection, Tissue Tolerance, and Na+ Exclusion
Source: Front Plant Sci. 2017 Apr 4;8:155. doi: 10.3389/fpls.2017.00155 (PMC5378810; doi:10.3389/fpls.2017.00155)
Supplement: Supplementary file 1 [file Image_1.pdf]

Supplementary materials

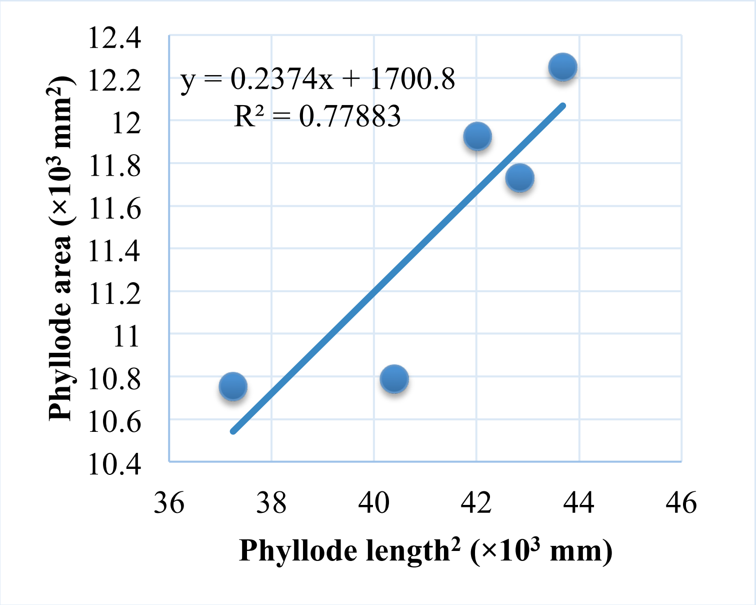

Supplementary Fig. 1. Relationship between phyllode length^2 (L^2) and phyllode area (PA) of *Acacia auriculiformis* plants grown under control conditions (n = 5 phyllodes). The projected phyllode area was calculated using the regression equation of "(0.2374+1700.8) L^2".

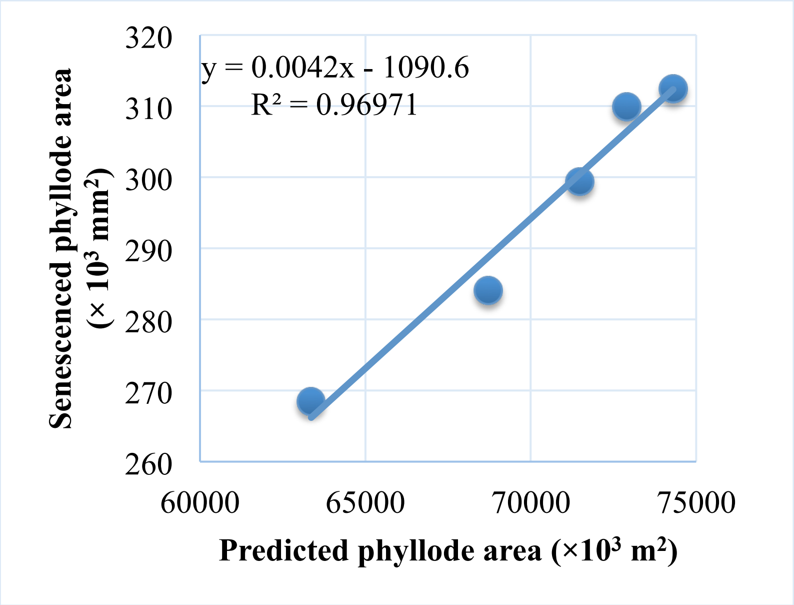

Supplementary Fig. 2. Relationship between predicted and senescenced phyllode areas of *Acacia auriculiformis* plants grown under control conditions (n = 5 phyllodes). The predicted area of natural senescence in salt-treated plants was calculated using the equation of "$y = 0.0042x - 1090.6$".
